# Unraveling the cross-talk between a highly virulent PEDV strain and the host via single-cell transcriptomic analysis

**DOI:** 10.1128/jvi.00555-25

**Published:** 2025-05-21

**Authors:** Yanan Wang, Yu Cheng, Shuai Wang, Dan Liu, Yueyi Gao, Jiaxuan Li, Yanping Jiang, Wen Cui, Xinyuan Qiao, Yijing Li, Li Wang

**Affiliations:** 1 College of Veterinary Medicine, Northeast Agricultural University 12430, Harbin, China; 2 Heilongjiang Key Laboratory for Animal Disease Control and Pharmaceutical Development, Harbin, China; 3 China Institute of Veterinary Drug Control 620909, Beijing, China; University of Michigan Medical School, Ann Arbor, Michigan, USA

**Keywords:** PEDV, single-cell RNA-seq, host-pathogen interactions, intestinal regeneration, lymphocyte infection

## Abstract

Porcine epidemic diarrhea virus (PEDV) causes severe intestinal damage and high mortality in neonatal piglets. The continuous emergence of new strains has brought new challenges to prevention and control. In this study, we isolated and characterized a prevalent PEDV virulent strain and analyzed 19,612 jejunal cells from PEDV-infected and control piglets using single-cell sequencing, revealing significant changes in cellular composition, gene expression, and intercellular communication. In response to PEDV infection, epithelial repair was enhanced through increased proliferation and differentiation of stem cells, transit-amplifying (TA) cells, and intestinal progenitor cells into enterocytes. Additionally, PEDV disrupted intercellular communication, compromising epithelial functionality while triggering immune responses, with IFN-γ and IL-10 signaling activation acting as critical regulators of immune balance and tissue homeostasis. Beyond enterocytes, viral genes were detected in various other cell types. Further experiments confirmed that PEDV could initiate replication in B and T lymphocytes but was unable to produce infectious progeny, with T cells additionally undergoing virus-induced apoptosis. These findings provide new insights into PEDV tropism, immune evasion, and epithelial repair, revealing complex host-pathogen interactions that shape disease progression and tissue regeneration, thereby contributing to a better understanding of enteric coronavirus pathogenesis.

**IMPORTANCE:**

The persistent circulation of porcine epidemic diarrhea virus (PEDV) poses a major threat to the swine industry, with emerging strains complicating prevention and control efforts. Currently, no effective measures completely prevent virus transmission, highlighting the need to understand PEDV-host interactions. In this study, we isolated a prevalent virulent strain and used single-cell sequencing to identify new PEDV-infected cell types and explore the complex interplay between the host and PEDV. These findings provide essential insights into viral pathogenesis and facilitate the design of targeted antiviral interventions.

## INTRODUCTION

Coronaviruses are enveloped positive-sense, single-stranded RNA viruses that are important pathogens in humans and vertebrates and pose a worldwide threat to public health security. CoVs can be divided into four genera that include *Alphacoronavirus, Betacoronavirus, Gammacoronavirus,* and *Deltacoronavirus* (1). Porcine epidemic diarrhea virus (PEDV), belonging to the *Alphacoronavirus* genus within the *Coronaviridae* family of the *Nidovirales* order, induces severe symptoms such as acute diarrhea, vomiting, dehydration, and high mortality in neonatal piglets, leading to substantial economic repercussions (2, 3). The continuous emergence of novel PEDV strains complicates prevention and control efforts, making the identification and characterization of these new variants, along with their pathogenic mechanisms, crucial for understanding the evolving threat and developing effective control strategies.

PEDV infection causes substantial damage to the intestinal epithelium, including villous atrophy and epithelial cell loss, which disrupts the intestinal immune environment and impairs barrier function (4–7). The intestinal epithelial barrier, consisting of epithelial, immune, and mesenchymal cells, acts as the primary defense against viral infections (8). In response to infection, the barrier activates immune cells, produces cytokines and antibodies to limit viral spread, and initiates epithelial repair to restore barrier integrity (9). A comprehensive understanding of the interactions between PEDV and its host is essential for developing effective therapeutic strategies. Although existing studies have provided valuable insights, they often average responses across large cell populations, thus overlooking the heterogeneity within tissue subsets. To better capture this complexity, high-resolution single-cell analysis is crucial for elucidating the diverse cellular responses to viral infection.

Single-cell RNA sequencing (scRNA-seq) has emerged as a powerful and unprecedented tool to explore cellular heterogeneity, offering deep insights into the molecular mechanisms underlying viral pathogenesis (10). Unlike traditional bulk RNA sequencing, scRNA-seq enables the examination of gene expression at the single-cell level, thereby revealing cellular diversity that is often masked in population-based analyses (11–14). In the context of viral infection, scRNA-seq offers a unique advantage by allowing the dissection of intricate interactions between individual cell types, providing a comprehensive view of cellular dynamics, immune activation, and the signaling pathways involved in both viral invasion and host defense. This high-resolution approach is crucial for identifying rare cell populations, uncovering previously unrecognized viral-host interactions, and ultimately improving our understanding of viral infection and immune responses at a more refined level.

In this study, we isolated a highly virulent PEDV strain from a pig farm in Heilongjiang Province experiencing a severe diarrhea outbreak, and pathogenicity assays confirmed its high virulence. To gain deeper insights into its pathogenic mechanisms, we performed single-cell sequencing on 19,612 individual jejunal cells from PEDV-infected and healthy piglets, aiming to obtain an unbiased understanding of the intestinal alterations induced by PEDV infection. Our findings reveal the changes in cell composition and activation, including the identification of previously unrecognized cell types that are newly infected during PEDV infection. We also highlight key signaling pathways involved in viral infection, immune response, and tissue repair. These results provide valuable insights into the mechanisms underlying PEDV pathogenesis and suggest potential therapeutic strategies to mitigate viral-induced intestinal damage.

## RESULTS

### A novel PEDV virulent strain was isolated from diarrhea piglets

**Fig 1:**
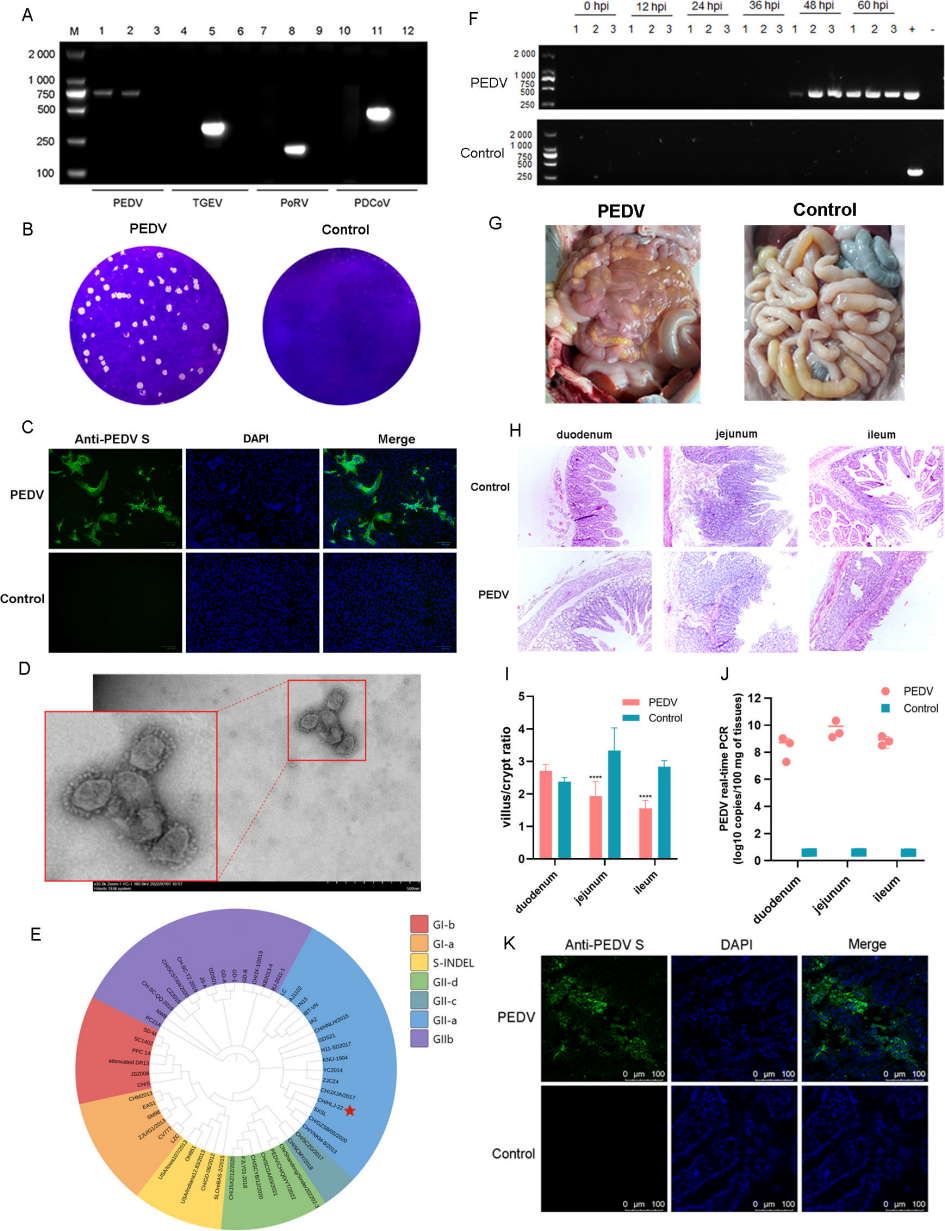
Isolation and identification of the CH/HLJ-22 strain. (**A**) Detection of PEDV by PCR in tissue samples from pig farms. (**B**) Plaque morphology of the isolated PEDV strain. (**C**) Cytopathic effects (CPEs) of PEDV in Vero cells. (**D**) Image of PEDV particles by electron microscopy. (**E**) Genotyping of the PEDV based on the S gene. (**F**) Detection of PEDV in feces in PEDV-infected and control piglets. (**G**) The pathogenicity of PEDV in 3-day-old piglets. (**H**) Histopathological examination of intestinal tissues from piglets infected with CH/HLJ-22 or treated with DMEM. (**I**) Ratio of villus height to crypt depth in different experimental groups across three intestinal segments. The ratio was calculated by measuring the villus height and crypt depth in tissue sections from the control and PEDV-infected groups in the duodenum, jejunum, and ileum. Significant differences between the groups are indicated by asterisks (*P* < 0.05). Data are presented as the mean ± SEM (*n* = 5 per group). (**J**) Viral copies in the small intestine tract tissues of the PEDV-infected and control piglets. (**K**) Immunofluorescent detection of PEDV antigen in the jejunum of PEDV-infected piglets.

Piglet intestine samples were collected from a farm with a remarkable outbreak of acute diarrhea, and PCR results revealed that PEDV was a diarrhea-causing pathogen (Fig. 1A). A PEDV strain was successfully isolated and propagated in Vero cells, and a pure PEDV line was established by isolating the viral population produced in a single plaque (Fig. 1B). A typical cytopathic effect (CPE), syncytium, caused by PEDV infection was observed in the presence of trypsin (Fig. 1C). Typical crown-like spikes of coronavirus particles were observed by electron microscopy (Fig. 1D). The isolated strain was designated as CH/HLJ-22, and its complete genome was submitted to GenBank (GenBank accession number PV449162.1). The complete genome of CH/HLJ-22 was obtained, and there was 91.5%–97.5% nucleotide sequence identity with those of the reference strains reported in GenBank (Table S1). Phylogenetic analysis of the S gene revealed that CH/HLJ-22 was clustered into GII-a (Fig. 1E).

The pathogenicity of PEDV was determined in piglets that exhibited typical clinical symptoms, including watery diarrhea and vomiting, from 36 h post-infection (hpi) until they died rapidly at 60 hpi. Simultaneously, viral shedding was consistently detected in the anal swabs of the infected group at 48–60 hpi (Fig. 1F). Clinical necropsies of the PEDV-infected piglets showed that the walls of their small intestines were thin, translucent, and contained watery fluid (Fig. 1G). Histopathological examination (HE) revealed severe villous atrophy and shedding of the intestinal villi in the duodenum, jejunum, and ileum (Fig. 1H). The ratio of villus height to crypt depth (villus:crypt) was calculated and shown in Fig. 1I. The highest viral load in the jejunum was confirmed by quantitative real-time PCR (qRT-PCR) (Fig. 1J). Immunofluorescence (IF) staining further confirmed abundant replication of PEDV in the jejunum (Fig. 1K); hence, the jejunum was subjected to single-cell sequencing.

### Single-cell transcriptome landscape of the piglet jejunum

**Fig 2:**
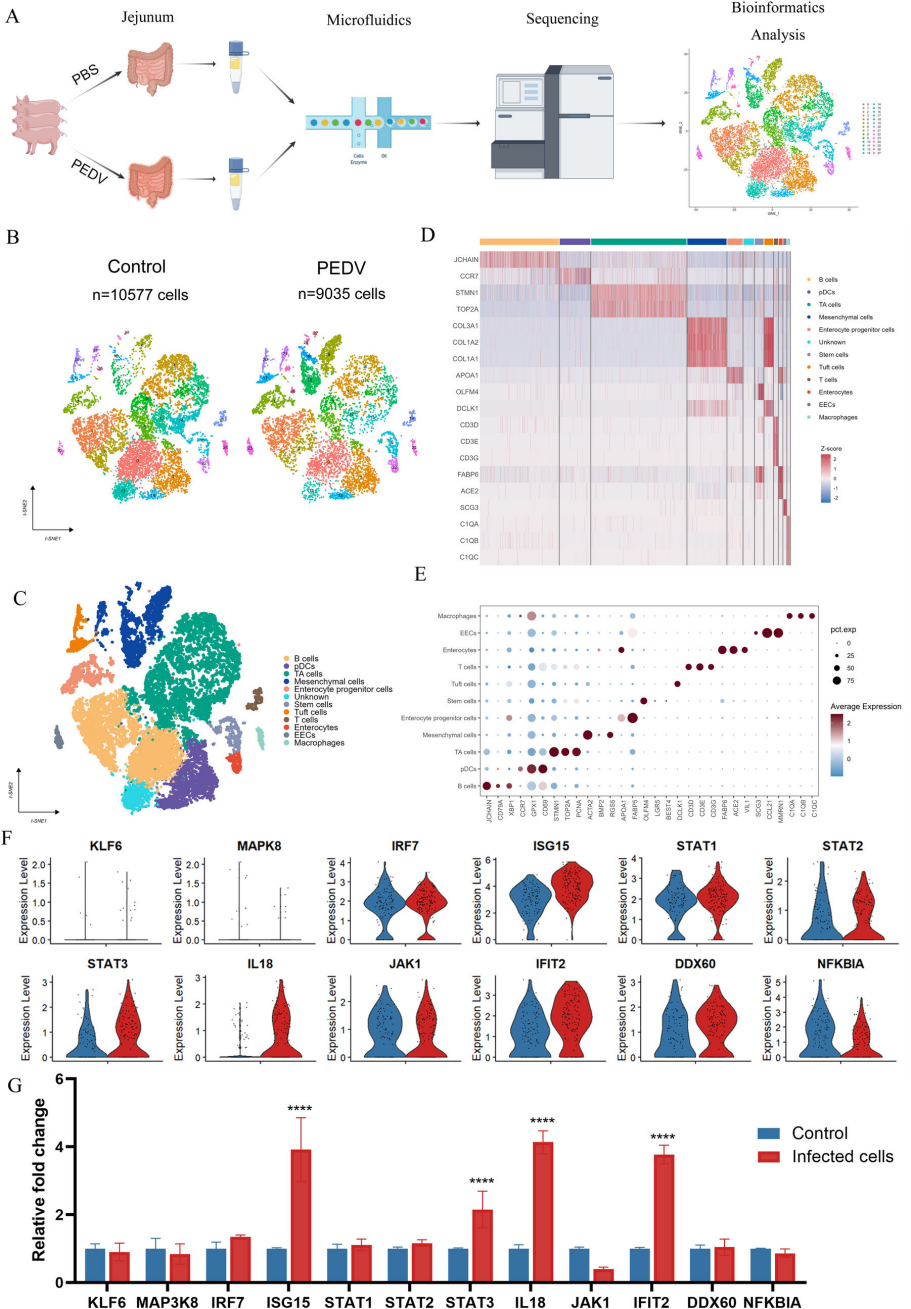
Single-cell transcriptomic analysis reveals the impact of PEDV on jejunal tissues in piglets. (**A**) Overview of the experimental design and bioinformatics analysis workflow. (**B**) tSNE of 19,612 intestinal cells from uninfected and infected piglets. (**C**) tSNE plot showing cell types identified in the control and PEDV-infected group. (**D**) Heatmap indicating the differential expression of genes specific to cell types in intestinal structural and immune cells. Colors ranging from blue to red indicate gene expression levels from low to high. (**E**) Dot plot showing the mean expression of marker genes used to classify cell clusters. The color intensity represents gene expression levels, whereas dot size indicates the fraction of cells expressing the gene within each cell type. (**F**) Vlnplot showing the expression levels of selected genes in enterocytes from control (blue) and PEDV-infected (red) groups, based on single-cell RNA-seq data. (**G**) qRT-PCR analysis of the same set of genes in porcine ileal epithelial cells (IPI-2I cells) confirmed their transcriptional changes upon PEDV infection. Results are shown as relative fold changes compared with controls. Data represent mean ± SD from three independent experiments. ****P < 0.0001 (unpaired two-tailed Student’s *t*-test)

A part of the jejunum of each piglet was randomly selected for single-cell sequencing, and the overview of the approach is provided in Fig. 2A. After cell filtration, 10,577 and 9,035 high-quality cells were obtained from the control and infected groups, respectively, and distributed into 28 clusters at 0.85 resolution (Fig. 2B; Fig. S1A through S1D). Eleven cell types were annotated on the basis of predominant markers, including enterocytes (FABP6, ACE2, and VIL1), enterocyte progenitor cells (APOA1 and FABP5), stem cells (OLFM4, LGR5, and BEST4), transit amplifying (TA) cells (STMN1, TOP2A, and PCNA), EECs (SCG3, CCL21, and MMRN1), tuft cells (DCLK1), macrophages (C1QA, C1QB, and C1QC), pDCs (CCR7, GPX1, and CD69), B cells (JCHAIN, CD79A, and XBP1), T cells (CD3D, CD3E, and CD3G), and mesenchymal cells (ACTA2, BMP2, and RGS5) (Fig. 2C through 2E). The cells clustered without a visible infection-specific pattern, indicating no drastic change in cell type. The transcriptomic changes were identified by GO analysis based on the differentially expressed genes (DEGs). The results showed that cellular functions related to the regulation of cell death, regulation of immune response, cellular response to viruses, and positive regulation of signal transduction were enriched in the comparison between the PEDV-infected and control groups (Fig. S2). To validate the reliability of the ScRNA-seq data, we selected a subset of representative DEGs and confirmed their expression changes using qRT-PCR, which showed consistent expression patterns with the sequencing results (Fig. 2F and 2G).

### PEDV infection promotes stem cell differentiation: a potential mechanism for self-repair in the small intestine

**Fig 3:**
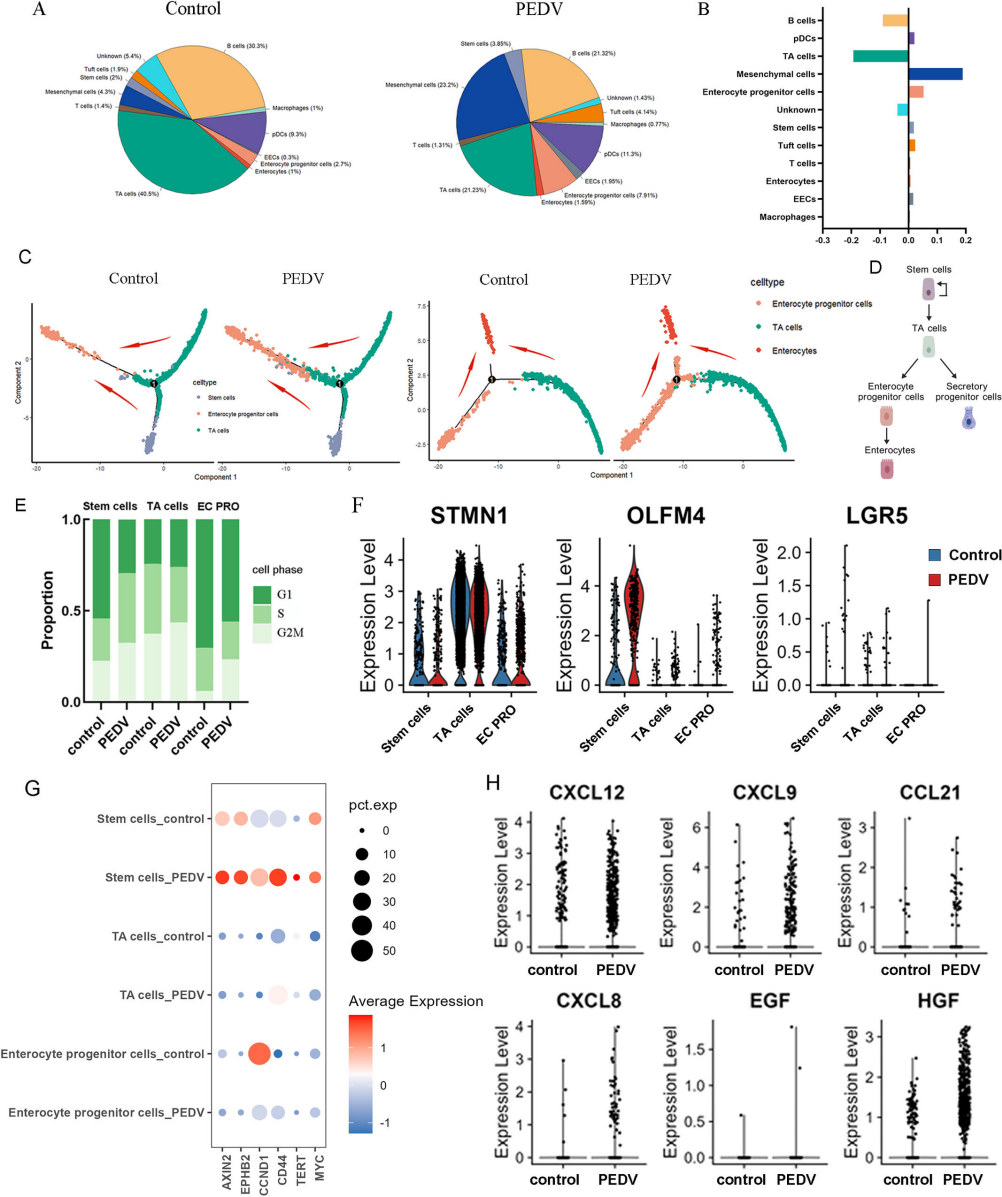
PEDV infection enhances stem cell differentiation. (**A**) Pie chart showing the proportion of each cell type in PEDV-infected and control groups. (**B**) Bar chart displaying the percentage difference in each cell type between infected and control groups. (**C**) Differentiation pseudotime trajectory analysis of stem cells, TA cells, and enterocyte progenitor cells, and the red arrow indicates the direction of differentiation. (**D**) This schematic diagram illustrates the differentiation trajectory. from stem cells to transit-amplifying (TA) cells and from enterocyte progenitor cells to enterocytes. (**E**) Histogram indicating the different cell-cycle quantity of stem cells, TA cells, and EC PRO, in PEDV infection and control groups. (**F**) Expression of cell cycle markers in the control and infected stem cells, TA cells, and EC PRO. (**G**) DotPlot indicates the expression of well-established WNT signaling pathway target genes in stem cells, TA cells, and EC PRO. (**H**) VlnPlot indicates the expression of chemokines and growth factors in the mesenchymal cells.

Although PEDV infection did not lead to the complete absence or emergence of any cell type, alterations in the composition ratios among various cell types were observed (Fig. 3A and B). The intestinal epithelium is the fastest self-renewing tissue where stem cells possessing robust proliferative and differentiation capabilities generate TA cells and progenitors, which then differentiate into mature functional cell types (15, 16). The pseudotime for the differentiation of stem cells, TA cells, and enterocyte progenitor cells (EC PRO) represented the transition of stem cells into TA cells, the subsequent differentiation of TA cells into enterocyte progenitor cells, and ultimately, the differentiation of all these precursor cells towards enterocytes (Fig. 3C). PEDV infection accelerated the differentiation process. Specifically, we have noted alterations in cells possessing the capacity to differentiate into intestinal lineages, including stem cells and TA (transit-amplifying) cells, and enterocyte progenitor cells (Fig. 3D). Further analysis revealed a notable increase in the percentage of cells in the G2M phase among the aforementioned cells (Fig. 3E), and the expression levels of proliferation-related genes such as STMN1, OLFM4, and LGR5 were upregulated (Fig. 3F), indicating strong cellular proliferation. Multiple pathways coordinate the orderly proliferation and differentiation of stem cells, among which Wnt signaling plays a pivotal role (17). The upregulation of the Wnt target gene expression, including Axin2, EPHB2, CCND1, CD44, TERT, and MYC in PEDV-infected cells, further highlights the activation of WNT signaling in response to infection (Fig. 3G). Mesenchymal cells play a vital role in tissue repair, regeneration, and the creation of a supportive microenvironment for epithelial renewal (18). In the context of PEDV infection, an increased proportion of mesenchymal cells with heightened expression of chemokines (CXCL12, CXCL9, CCL21, and CXCL8) and growth factors (EGF, HGF) was observed, suggesting that mesenchymal cells may promote epithelial repair by creating a regenerative microenvironment (Fig. 3H). These findings indicate that stem cells repair PEDV-induced intestinal damage by enhancing their proliferation and differentiation, with WNT signaling playing a critical regulatory role. Furthermore, mesenchymal cells likely enhance this process by fostering a supportive microenvironment, collectively promoting intestinal epithelial regeneration and repair.

### Cell-cell communication network in control and PEDV-infected intestine

**Fig 4:**
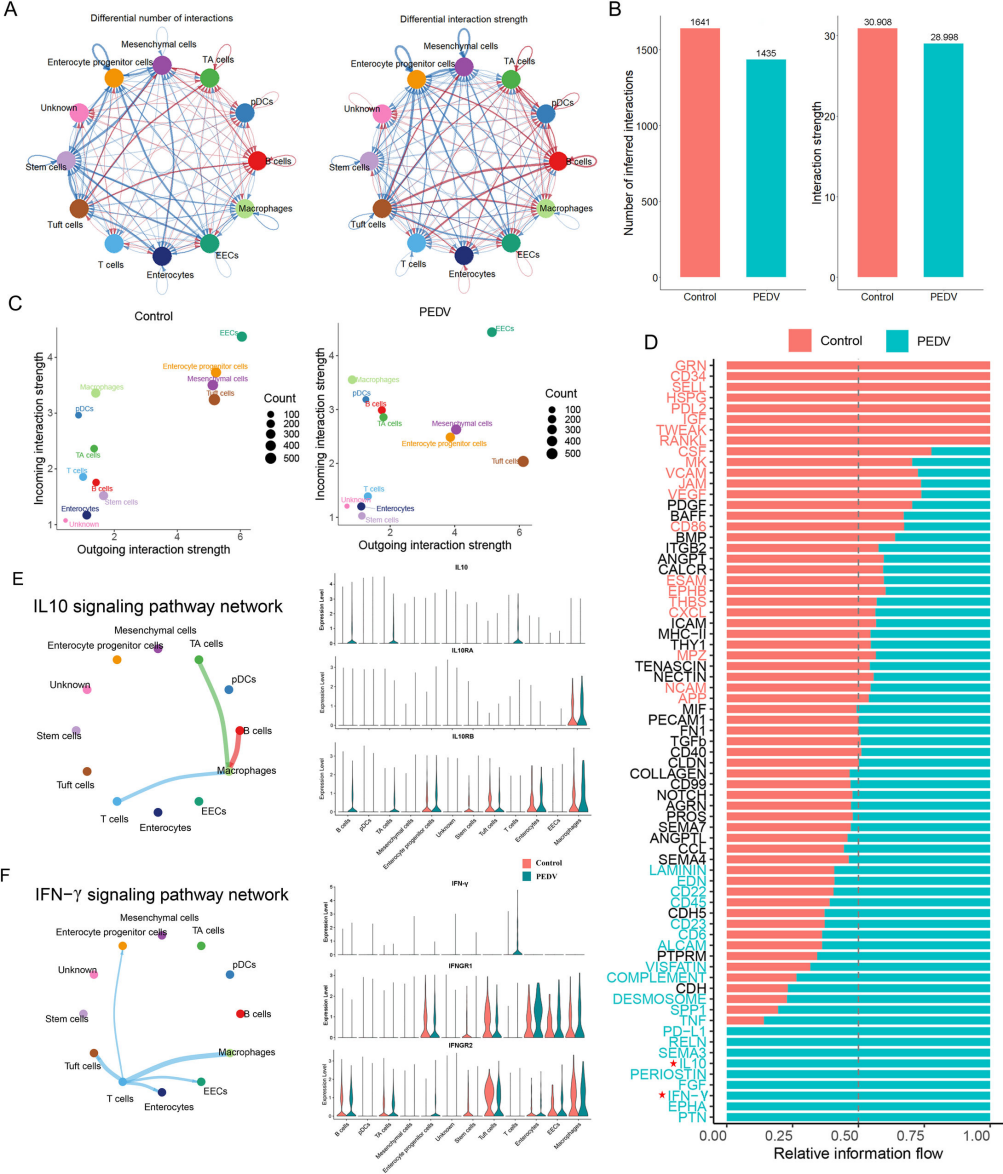
Cell-cell communications between control and PEDV-infected intestine. (**A**) Interaction network count plot (left) and interaction weight plot (right) of each cell type in infected compared and groups. Thicker lines indicate a higher number of interactions and greater interaction strength between cell types. Blue lines represent reduced communication in the PEDV-infected group, whereas red lines indicate increased communication relative to the control group. (**B**) Comparison of the total number of cell communication interactions and the intensity of interactions. (**C**) Scatter plot showing the primary signaling sources and targets in PEDV-infected and control groups. Colors represent cell types, and dot size reflects the number of significantly expressed receptor-ligand interactions. (**D**) Ranking of significant signaling pathways based on differences in overall information flow within inferred networks between PEDV infections. (**E**) Inferred IL-10 intercellular communication network shown in a circular plot (left). Thicker lines indicate a higher number of interactions and greater interaction strength between cell types. Expression levels of IL-10 pathway genes in each cell type are compared between PEDV-infected (blue) and control (red) groups. Normalized expression levels are visualized in a violin plot (right). (**F**) Inferred IFN-γ intercellular communication network shown in a circular plot (left). Thicker lines indicate a higher number of interactions and greater interaction strength between cell types. Expression levels of IFN-γ pathway genes in each cell type are compared between PEDV-infected (blue) and control (red) groups. Normalized expression levels are visualized in a violin plot (right).

The analysis of intercellular communication revealed a decline in both the number and intensity of interactions following PEDV infection, suggesting that molecular signaling and interactions were significantly disrupted during viral infection (Fig. 4A and B). The input signaling intensity of tuft cells, enterocyte progenitor cells, and mesenchymal cells decreased, suggesting a potential impairment of epithelial functionality. Conversely, macrophages, dendritic cells, and B cells exhibited increased output signaling intensity, indicating enhanced immune cell activation post-infection (Fig. 4C). The enhanced interactions of ligand-receptor pairs such as PTPRC-CD22, CD74-CXCR4, and CD99-CD99 following infection suggest strengthened signaling after infection, accelerated recruitment and migration, and dynamic remodeling of the immune microenvironment (Fig. S3). To further explore changes in signaling pathways, key pathways were ranked based on differences in information flow between the PEDV-infected and control groups. Although most pathways remained active in both conditions, 24 of 71 exhibited significant dynamic shifts following infection. These pathways were primarily involved in antiviral responses, cell adhesion, inflammation, apoptosis, and tissue repair (Fig. 4D). Notably, IFN-γ and IL-10 were identified as core immunoregulatory pathways uniquely activated during PEDV infection (Fig. 4E and F). Detailed analysis of IFN-γ and IL-10 signaling during PEDV infection, based on ligand-receptor expression, revealed that T cells, as major donors of IFN-γ, highly expressed the IFN-γ gene. This likely enhanced the antiviral activity of enterocytes or macrophages through the receptor IFNGR1. Similarly, IL10 was secreted by activated T cells and B cells, potentially suppressing pro-inflammatory cytokine expression in macrophages.

### PEDV tropism may not be exclusively restricted to enterocytes

**Fig 5:**
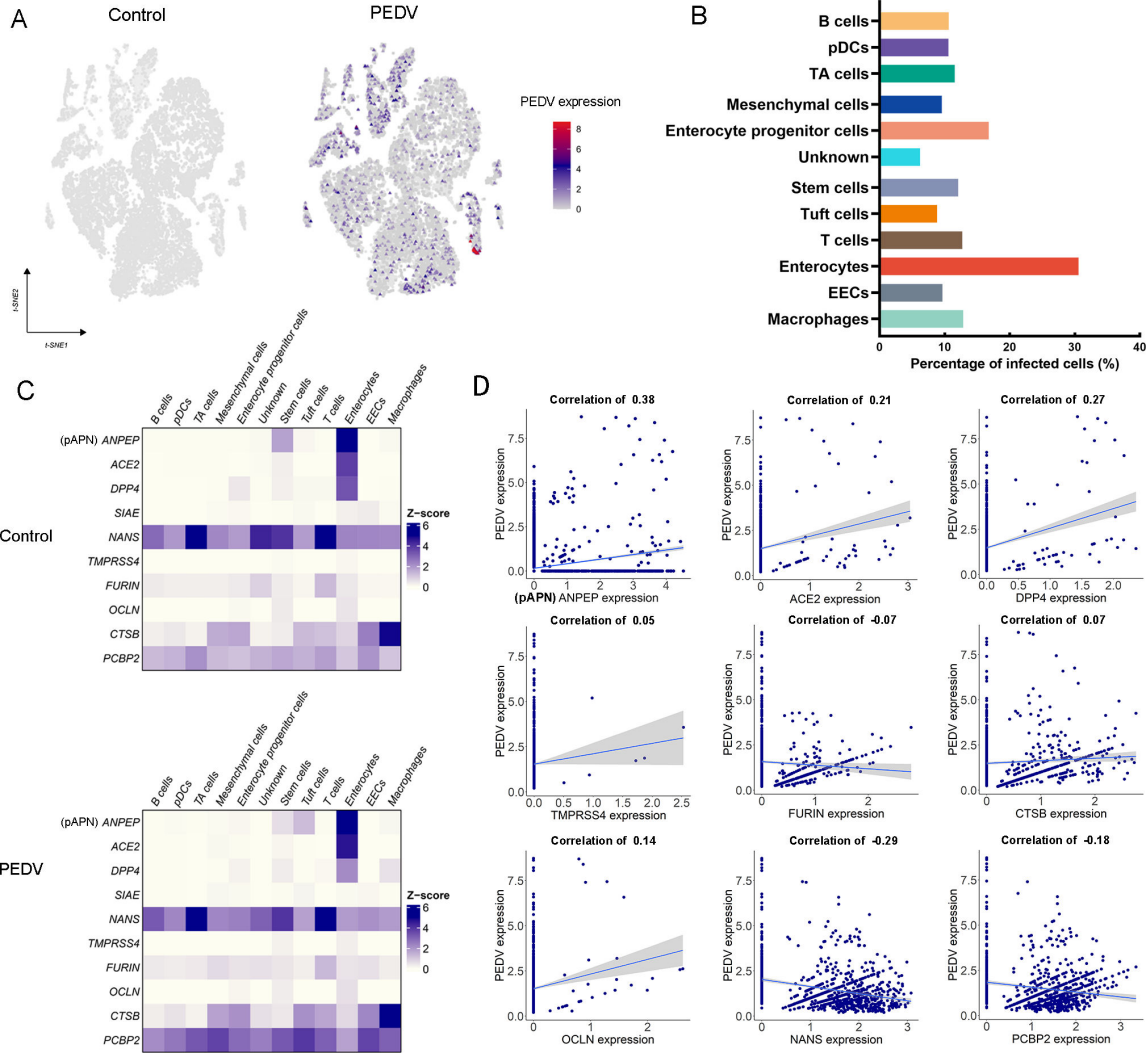
Determinants of cell tropism of PEDV in the porcine intestinal tract. (**A**) tSNE visualization of scRNA-seq data from PEDV-infected intestinal cells. Infected cells are marked as triangles, with colors representing the corrected, targeted, and normalized expression levels of PEDV based on scRNA-seq data. (**B**) Proportion of PEDV-infected cells across different cell types. (**C**) Heatmap showing the expression levels of coronavirus receptors in control and PEDV-infected groups. (**D**) Correlation analysis between PEDV gene expression and coronavirus receptor expression across all infected cells.

In total, 1,022 PEDV-positive cells were identified in the PEDV-infected group by aligning the sequencing reads to the PEDV genome (Fig. 5A). It is noteworthy that, in this study, although epithelial cells remain the primary target of PEDV infection, PEDV genes have also been detected at varying levels in other cell types, including immune cells (B cells, pDCs, T cells, and macrophages), TA cells, mesenchymal cells, enterocyte progenitor cells, stem cells, et al. (Fig. 5B). Receptors and proteases of host cells play crucial roles in the cell tropism and replication of the viruses, so the coronavirus-related receptors (ANPEP (also known as pAPN), ACE2, DPP4, SIAE, and NANS), and key cellular proteases (TMPRSS4, OCLN, FURIN, CTSB, and PCBP2) that had been reported were analyzed in this study (19–22). The results showed that the receptors pAPN, ACE2, and DPP4 were significantly highly expressed in enterocytes (Fig. 5C), and the association between the above receptors and the susceptibility of PEDV-infected cells was verified by the correlation analysis (Fig. 5D). There was no markedly differential pattern of key cellular proteases expression among these types of cells (Fig. 5C). These results indicate that the extensive expression of relevant important receptors is a pivotal factor rendering enterocytes the primary target for PEDV infection. However, further research is necessary to conclusively determine the susceptibility of other cell types to PEDV infection and identify the associated receptors involved.

### PEDV is capable of infecting B cells yet fails to establish effective replication or affect B cell differentiation

**Fig 6:**
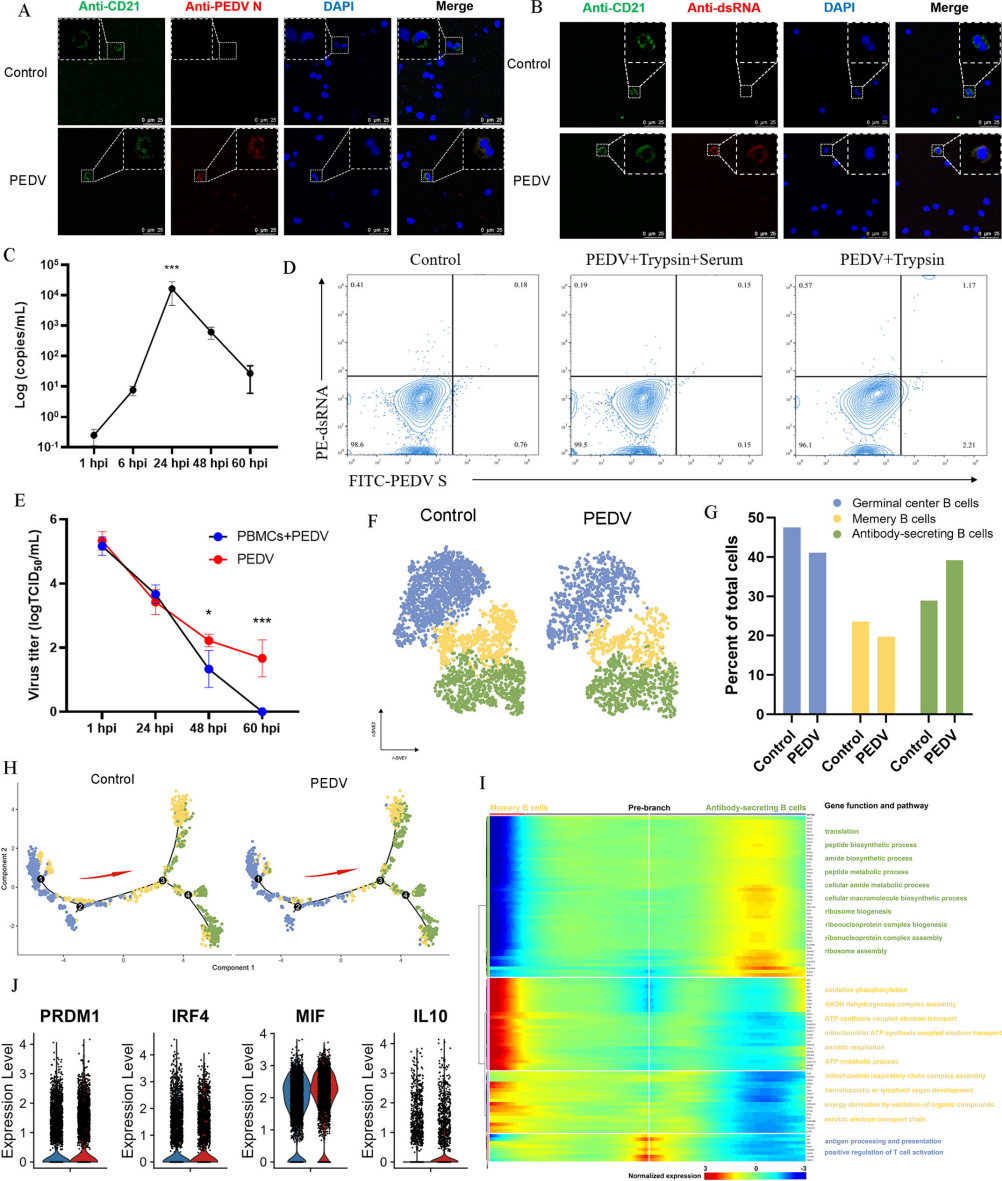
PEDV is capable of infecting B cells yet fails to establish effective replication or affect B cell differentiation. (**A**) Immunofluorescence staining for PEDV N protein in B cells infected with PEDV at 24 hpi. Scale bar, 25 µm. (**B**) Immunofluorescence staining for dsRNA in B cells infected with PEDV at 24 hpi. Scale bar, 25 µm. (**C**) Quantification of PEDV subgenomic RNA levels in PEDV-infected PBMCs at different time points post-infection. (**D**) Infection rates of B cells at 24 hpi were determined by FACS analysis in the presence or absence of serum. (**E**) Replication curve of PEDV with mean of virus titers (in TCID_50_/mL) in supernatants from cultures of PBMCs. (**F**) Subclustering of B cells in the PEDV infection and control group. (**G**) The proportion of B cell subsets in the PEDV infection and control groups. (**H**) Pseudotime trajectory analysis of B cell differentiation. Predicted secretory lineage cells include GCBs, ASCs, and MBCs, with the red arrow indicating the direction of differentiation. (**I**) The differentially expressed genes (rows) along the pseudotime (columns) of MBCs and ASCs clusters hierarchically into three profiles. The representative gene functions and pathways of each profile are presented on the right. (**J**) The differential expression levels of the PRDM1, IRF4, MIF, and IL10 genes in the B cells.

Given that PEDV genomic material was detected within B cells and that the number of B cells decreased (Fig. 5B and 3B), we infected porcine peripheral blood lymphocytes (PBMCs) with PEDV to confirm whether the virus can infect B cells within this population. The presence of PEDV N Protein, double-stranded RNA (dsRNA), and subgenomic RNA (sgRNA) in B cells following incubation with PEDV confirmed that PEDV infection and replication occurred within these cells (Fig. 6A through C). As antigen-presenting cells (APCs), B cells internalize antigens and process them for presentation to T cells (23). PEDV infection relies on trypsin activation, which is effectively blocked by serum (24). To determine whether PEDV enters B cells via direct infection or passive antigen uptake, we analyzed PEDV S protein and dsRNA expression in the presence or absence of serum. The results showed that PEDV S protein and dsRNA increased only in the serum-free group, whereas there was no significant difference between the serum-containing group and the control group (Fig. 6D). These results indicate that PEDV enters B cells through direct infection rather than being taken up as antigens. Multiple assays confirmed that PEDV initiates replication in B cells; we next investigated whether these cells produce infectious viral progeny. We cultured PEDV-infected PBMCs for 60 h, and a group containing only PEDV virus particles was set up as a control. Although infectious viruses were detected in the supernatants of the PEDV-infected PBMCs group at early time 12-48 hpi (likely reflecting input virus), no infectious PEDV was detected at 60 h. By contrast, the virus was still detectable in the control group at 60 h (Fig. 6E). These results demonstrated that PEDV, which infected PBMCs including B cells, was incapable of producing viable progeny viruses. Although PEDV cannot effectively replicate within B cells, whether its entry into B cells affects their function still requires further exploration. The analysis results of B cell subpopulations showed that B-lineage lymphocytes were categorized into three subsets based on cell surface markers: antibody-secreting B cells (ASCs), germinal center B cells (GCBs), and memory B cells (MBCs) (Fig. 6F). The data revealed a significant decrease in both GCBs and MBCs, whereas ASCs showed a notable increase (Fig. 6G). Trajectory analysis of these subsets indicated that GCBs were positioned at the beginning of the trajectory, ASCs at the terminal stage, and MBCs were distributed across the entire lineage (Fig. 6H). Gene Ontology (GO) analysis of these subsets highlighted that GCBs were enriched in processes related to antigen processing, presentation, and T cell activation regulation, whereas ASCs showed high expression levels of genes involved in peptide metabolism, ribonucleoprotein complex biogenesis, and ribosome assembly, reflecting the increased demand for antibody production caused by PEDV infection (Fig. 6I). Additionally, markers such as PRDM1, IRF4, MIF, and IL10, which are key regulators of B cell activation and immunoglobulin synthesis, were upregulated (Fig. 6J). In summary, our findings demonstrate that PEDV can infect and initiate replication in B cells but fails to support productive viral replication. Additionally, PEDV infection promotes ASCs expansion, indicating an enhanced differentiation of B cells toward antibody-secreting cells.

### PEDV infection can impair T cell function and induce its apoptosis

**Fig 7:**
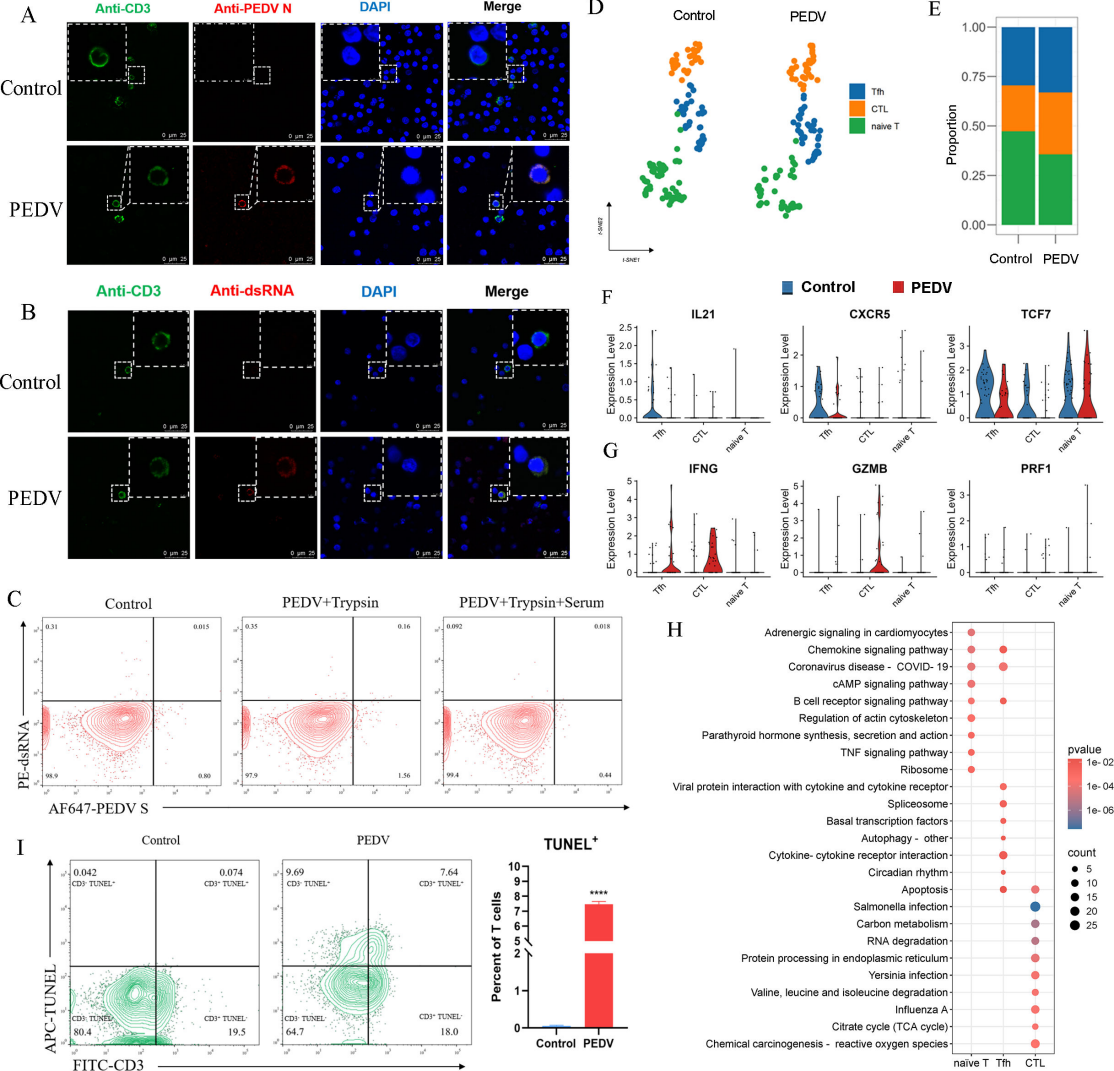
The functional alterations in T cells from intestinal tissues during PEDV infection. (**A**) Immunofluorescence staining for PEDV N protein in T cells infected with PEDV at 24 hpi. Scale bar, 25 µm. (**B**) Immunofluorescence staining for dsRNA in T cells infected with PEDV at 24 hpi. Scale bar, 25 µm. (**C**) Infection rates of T cells at 24 hpi were determined by FACS analysis in the presence or absence of serum. (**D**) Subclustering of T cells in the small intestine. (**E**) The proportion of T cell subtypes in the PEDV infection and control groups. (**F**) The differential expression levels of the genes IL21, CXCR5, and TCF7 in the T cell subsets. (**G**) The differential expression levels of the genes IFNG, GZMB, and PRF1 in the T cell subsets. (**H**) The KEGG signaling pathway enrichment in T cell subclusters from PEDV-infected compared with the control group. (**I**) FACS analysis of apoptosis in PEDV-infected T cells. The apoptosis rate is visualized in a scatter plot (left) and quantified in a histogram (right).

Given the detection of PEDV genomic RNA in T cells (Fig. 5B), the infection of PEDV in T cells was further investigated in this study. The results of IFA and flow cytometry showed the presence of PEDV N and double-stranded RNA (dsRNA) in T cells, indicating the PEDV infection and active replication in these cells (Fig. 7A through 7C). Since T cells cannot phagocytose antigens, PEDV N and dsRNA became undetectable after the addition of serum, indicating that the process of PEDV infecting T cells *in vitro* required the involvement of trypsin (Fig. 7C). Based on the results mentioned above (Fig. 6E), we can conclude that infectious progeny failed to be produced in PEDV-infected T cells as well. To explore the effects of PEDV infection on T cells, we analyzed T cell subpopulations based on marker gene expression. T cells were classified into naïve T cells, T follicular helper (Tfh) cells, and cytotoxic T lymphocytes (CTL) (Fig. 7D). Following infection, the proportion of naïve T cells decreased, whereas Tfh cells and CTL showed a relative increase, which may result from enhanced differentiation of naïve T cells into Tfh cells and CTL caused by PEDV infection (Fig. 7E). IL21, CXCR5, and TCF7 are key regulators of Tfh cell function, which is associated with guiding Tfh cell migration to germinal centers, promoting B cell differentiation and antibody class switching, and controlling the development and function of Tfh cells (25). The decreased expression of IL21, CXCR5, and TCF7 suggests a potential impairment in the ability of Tfh cells to support B cell immune response (Fig. 7F). In CTL, the expression of key antiviral mediators, such as IFN-γ and granzyme B (GZMB), was upregulated, whereas the level of perforin (PRF1), essential for the killing of infected cells, remained unchanged (Fig. 7G), suggesting that although CTL responses were activated, their cytotoxic function may be weakened. KEGG pathway analysis further revealed significant activation of apoptosis-related pathways in both Tfh and CTL cells (Fig. 7H). Consistently, flow cytometry results showed an average 11.9% increase in T cell apoptosis following PEDV infection compared to the control group (Fig. 7I), indicating that the virus can actively promote T cell death. In summary, PEDV can infect T cells and initiate replication, but it does not complete a productive replication cycle. Moreover, PEDV infection may impair T cell function and promote T cell apoptosis.

## DISCUSSION

The outbreak of PEDV has resulted in significant economic losses in the global pig industry since 2010, which primarily causes acute watery diarrhea and high mortality in suckling piglets (26, 27). Despite intensified biosecurity measures and iterative updates to vaccine formulations, phylogenetically distinct emerging PEDV variants persistently cause breakthrough infections (28). Here, a highly pathogenic PEDV strain, CH/HLJ-22, was successfully isolated from a farm in Heilongjiang province with a diarrhea outbreak, and it was found to cause piglet mortality within 60 h. The phylogenetic analysis revealed that the PEDV CH/HLJ-22 clustered within the GIIa subgroup, confirming the persistent circulation of GIIa PEDV. This finding indicates that although novel GIIb, GIIc, and GIId strains are increasingly emerging, GIIa strains retain critical importance in regional outbreaks and continue to exert substantial impacts on swine farming operations (29–31). To explore the pathogenic mechanism of the PEDV strain isolated in this study, we employed single-cell sequencing to characterize cellular dynamics within infected porcine small intestine tissues. This comprehensive profiling not only revealed critical alterations in host cellular landscapes but also illuminated the complex host-pathogen interplay, particularly highlighting two counterbalancing forces during viral invasion, the intestinal epithelium’s regenerative resilience and the host’s orchestrated immune counterattack. These findings collectively demonstrate the dynamic battlefield where viral pathogenesis and host defense mechanisms engage in a molecular arms race.

A key strength of single-cell RNA sequencing in the study of viral pathogenesis lies in its capacity to dissect individual cell types within complex tissues, thereby providing precise insights into the specific roles each cell plays in the infection process (32, 33). In our results, we observed overall changes in the proportions of intestinal cell types following PEDV infection, particularly a marked reduction in the proportion of transit-amplifying (TA) cells. TA cells are generated from stem cells and then rapidly proliferate and finally differentiate into specialized epithelial cells (34, 35). Pseudotime trajectory analysis revealed enhanced differentiation trajectories from stem cells and TA cells through enterocyte progenitor cells toward mature intestinal enterocytes post-infection, suggesting that the reduction in TA cells may be due to their rapid differentiation into intestinal enterocytes following viral infection, thereby maintaining intestinal tissue functionality. A similar phenomenon of reduced TA cells after infection was also observed during rotavirus infection (36). Although stem cell differentiation increased, the proportion of stem cells remained stable, which may be attributed to their robust self-renewal capacity (37, 38). We subsequently analyzed the cell cycle, expression of proliferation-related genes, and the Wnt pathway, which plays a critical role in stem cell self-renewal (39). The results showed an increase in the proportion of stem cells in the S and G2/M phases, upregulation of proliferation-related genes, and activation of the Wnt pathway, evidenced by the elevated expression of Wnt target genes such as AXIN2, EPHB2, CCND1, CD44, TERT, and MYC following infection. These findings indicate that self-renewal of stem cells was enhanced, which is consistent with our hypothesis. In addition, we also observed that the proportion of mesenchymal cells increased notably after PEDV infection, accompanied by upregulated expression of growth factors such as CXCL12, EGF, and HGF. As key regulators of the epithelial microenvironment, mesenchymal cells maintain intestinal epithelial homeostasis by modulating the self-renewal, proliferation, and differentiation of intestinal stem cells through the secretion of these factors (39–41). These results demonstrate that PEDV infection induces dynamic changes within the intestinal crypt, characterized by stem cell proliferation, accelerated TA cell differentiation, and mesenchymal cell-mediated enhancement of epithelial repair. The interplay between these mechanisms underscores a highly coordinated response of the intestinal epithelium to viral infection, and targeting the self-renewal and differentiation of stem cells and mesenchymal signaling may represent a novel therapeutic approach for mitigating virus-induced intestinal damage and promoting recovery.

The maintenance of intestinal homeostasis relies on the precise coordination of intercellular signaling. We analyzed interactions among different cell types in intestinal tissues, constructing a global map of cell-to-cell communication. Our analysis revealed a reduction in both the number and intensity of intercellular interactions following PEDV infection, suggesting that viral infection disrupts intercellular communication and particularly affects epithelial cell function. Several signaling pathways were uniquely activated following PEDV infection, primarily those involved in immune regulation, cell migration, tissue repair, angiogenesis, and proliferation, all of which play essential roles in viral infections and tissue homeostasis (42–47). Among them, IFN-γ and IL-10 signaling were particularly notable for their opposing yet complementary functions in immune modulation. IFN-γ enhances antiviral immunity by promoting immune activation and viral clearance, whereas IL-10 regulates inflammation to prevent excessive immune responses and tissue damage (44). In this study, IFN-γ receptor expression was upregulated in immune and epithelial cells after infection, suggesting an enhanced response that may activate the JAK-STAT pathway and induce interferon-stimulated genes, reinforcing antiviral defenses (48). Meanwhile, increased IL-10 receptor expression in macrophages, enterocytes, and progenitor cells implies a regulatory role in suppressing excessive immune activation and mitigating potential tissue damage. Additionally, consistent with the elevated IL-10 signaling activity, our analysis also revealed an increased proportion of M2 macrophages in intestinal tissues following PEDV infection (Fig. S4). Given the anti-inflammatory role of M2 macrophages, this shift may represent a host strategy to counteract virus-induced inflammation, maintain tissue integrity, and interfere with viral replication (49). These findings indicate that PEDV disrupts intercellular communication, whereas the host simultaneously mounts an antiviral response and engages mechanisms to maintain homeostasis, highlighting a dynamic interplay between viral disruption and the host’s regulatory adaptations.

An additional advantage of single-cell sequencing in studying viral pathogenesis is its capacity to trace viral RNA at the single-cell level, providing direct insights into the spectrum of susceptible cell types and potentially identifying new targets of viral infection (50). Previous studies have demonstrated that PEDV primarily infects and replicates in enterocytes at the villus tip, with low-level infection detected in goblet cells, pDCs, and macrophages (51–53). Our findings both support and extend these studies. A broader range of potential PEDV target cells, including enterocyte progenitor cells, stem cells, TA cells, EECs, tuft cells, B cells, T cells, and mesenchymal cells, were identified in this study, indicating that PEDV may infect a more diverse set of cell types in the gut than previously recognized. The varying infection rates across these cell types suggest differential susceptibilities, prompting further investigation into the determinants of PEDV tropism. Most reported CoV receptors and key proteases exhibited weak correlations (r < 0.3), whereas ANPEP (pAPN), a reported PEDV receptor (54), exhibited a moderate correlation (r = 0.38), not a high correlation. These data suggest that additional host factors may contribute to PEDV infection in different cell types. Similarly, the lack of differential expression of key proteases across various cell types suggests that protease expression alone does not strongly dictate PEDV susceptibility, reinforcing the idea that receptor availability may be a more critical factor. These findings underscore the complexity of PEDV cell tropism and suggest that while enterocytes remain the primary targets, viral entry into non-enterocyte populations may involve additional, yet unidentified, host factors, warranting further investigation.

Recent studies have revealed that CoVs, including SARS-CoV-2, MERS-CoV, and SARS-CoV, not only target epithelial cells but also directly infect various immune cell types (55–58). Consistent with these findings, our results revealed that PEDV exhibits tropism for both B and T cells *in vitro*, as evidenced by the detection of dsRNA, a hallmark of viral replication (56, 59), indicating that PEDV initiates replication in both B and T cells. However, in the presence of serum, infection was completely abolished, suggesting that the virus infects B cells by direct infection rather than passive antigen uptake. Interestingly, although PEDV initiates replication in both B and T cells, no progeny virions were detected in co-culture systems, indicating an abortive infection that prevents viral dissemination. A similar phenomenon has been observed for SARS-CoV-2, which initiates replication in monocytes but does not release infectious virions into the supernatant (60). Although these immune cells may not support efficient viral replication, they can serve as a "Trojan Horse" by harboring viral components within intracellular, thus shielding the virus from immune recognition and facilitating its dissemination to distant organs, as supported by studies on PEDV and Human Immunodeficiency Virus (HIV), which have shown that immune cells can transport viral particles and aid in viral spread (61–63). Additionally, we observed a notable reduction in intestinal B cell populations following PEDV infection, which could result from multiple factors. Our data revealed an increased proportion of antibody-secreting cells (ASCs) after infection, supporting the possibility that at least part of this substantial reduction may be due to antigen-driven B cell homing from the gut mucosa to lymphoid compartments. Furthermore, our results reveal that PEDV infection induces apoptosis in T cells, which may function as a double-edged sword in modulating host immune responses. Although apoptosis may help eliminate viruses and limit viral spread, excessive T cell death can impair adaptive immunity by reducing antigen-specific T cells, weakening the host’s antiviral response. These findings highlight the complex interplay between PEDV and the host immune system; understanding these mechanisms may offer valuable insights into antiviral strategies and therapeutic targets for PEDV and other coronaviruses.

In summary, the interaction between PEDV and its host is a dynamic and delicate balance. The virus continuously challenges the host, whereas the host defends itself through immune responses and tissue repair mechanisms. This ongoing interplay, where the virus attempts to evade host defenses and the host adapts to strengthen its response, ultimately determines the outcome of the infection. Understanding this balance is critical for developing targeted therapeutic strategies. Detailed study of these interactions could reveal new therapeutic targets, enhance disease management, and improve the ability to control viral outbreaks in the future.

## MATERIALS AND METHODS

### Cells and reagents

The Vero cells (ATCC CCL-81) and IPI-2I (porcine ileal epithelial cells) were cultured in Dulbecco’s modified Eagle’s medium (DMEM) supplemented with 10% fetal bovine serum (Gibco) at 37°C and 5% CO₂. The virus isolated from samples was identified as PEDV by N gene-based reverse transcription PCR (RT-PCR). Mouse anti-PEDV monoclonal antibodies (mAbs) were prepared in our lab. Mouse anti-porcine CD3ε-FITC and mouse Anti-Porcine CD21-AF647 were purchased from SouthernBiotech. R-PE Conjugation Kit/R-PE Labeling Kit, FITC Conjugation Kit/FITC Labeling Kit, and Alexa Fluor 647 Conjugation Kit were purchased from Abcam. dsRNA (J2) Mouse mAb was purchased from CST.

### Isolation and identification of PEDV

The clinical samples (small intestine tissues) used in this study were collected from a pig farm in Heilongjiang, China. The small intestines of piglets exhibiting diarrhea were processed into a suspension and subsequently filtered through a 0.22 µm pore-sized cellulose acetate filter. Vero cell monolayers were seeded in 12-well plates and then inoculated with the suspension in the presence of 25 µg/mL trypsin at 37 ℃ under 5% CO_2_ for 1 h. Following virus adsorption, the cells underwent two washes with sterile phosphate-buffered saline (PBS; pH 7.2) and were cultured with DMEM containing 6 µg/mL trypsin. Cell harvesting took place once the cytopathic effect (CPE) exceeded 80%, and virus replication in Vero cells was assessed through PCR and an indirect immunofluorescence assay (IFA). Subsequently, the viruses were plaque-purified and propagated in Vero cells. Briefly, after initial infection, cell culture supernatants were harvested and subjected to serial dilution. The diluted viral suspensions were then inoculated onto confluent monolayers of Vero cells and overlaid with agarose-containing medium to allow for plaque formation. After 2 days of incubation, individual plaques were isolated using sterile pipette tips and transferred to fresh Vero cell cultures for amplification. This plaque purification process was repeated at least three times to ensure clonal purity of the virus. The purified viral stocks were then propagated in Vero cells and stored at −80  °C until use. The morphology of the virion was examined using transmission electron microscopy, and the complete genome of the virus was sequenced. Phylogenetic analysis based on the S gene was then conducted, comparing the isolated PEDV with other reference PEDV strains listed in Table S1.

### Immunofluorescence staining (IFA)

To prepare jejunum tissue sections for histology, small intestinal tissue was initially fixed with a 4% paraformaldehyde/PBS solution at 4°C overnight. Subsequently, the fixed tissue was transferred to 70% ethanol before undergoing paraffin embedding and sectioning. The resulting slides were then deparaffinized in HistoClear and rehydrated through successive ethanol baths. Following these steps, the sections were incubated with the corresponding primary antibodies overnight at 4°C, followed by incubation with secondary antibodies diluted in a blocking solution for 1 h at 37°C. Nuclei were identified by incubating the sections with DAPI for 10 min at room temperature. Finally, confocal laser scanning microscopy was employed for the observation of tissue slices.

### Hematoxylin and eosin (HE) analyses

To investigate the pathological changes in PEDV-infected intestines, small intestine sections were subjected to HE staining. The tissues were first immersed in xylene and alcohol, followed by hematoxylin staining for 10 min. Subsequently, the sections were stained with eosin for 20 min, re-immersed in alcohol and xylene, and finally mounted with synthetic resin.

### Animal experiments and Sc-RNA sample preparation

Piglets at 3 days of age were analyzed for maternal antibodies by ELISA, and other piglet diarrhea viruses, including porcine rotavirus (PoRV), porcine transmissible gastroenteritis virus (TGEV), and porcine delta coronavirus (PDCoV), were detected by RT-PCR. Six piglets were randomly divided into two groups. For infection, piglets were orally gavaged with 5 mL of isolated virus (10^6.33^ TCID_50_/mL), and the control group was orally administered virus-free cell culture medium. All piglets were sampled at the same time point (60 hpi), when the infected piglets succumbed to disease, and the control piglets were euthanized. Viral loads in the duodenum, jejunum, and ileum of piglets were detected using qRT-PCR. Jejunal tissues (approximately 1 cm) were immediately flushed with cold Dulbecco’s phosphate-buffered saline (DPBS) and placed in preservation solution (Miltenyi Biotec). For single-cell sequencing experiments, the small intestines from three piglets were combined and treated as one sample. Jejunal tissues were dissociated into single-cell suspensions according to the instructions for the Multi Tissue Dissociation Kit 1 (Miltenyi Biotec). Trypan blue staining and hemocytometry were used to examine cell viability.

### Single-cell library preparation and sequencing

Single-cell libraries were generated using a 10× Chromium NextGEM Single Cell 30 reagent kit (v3.1) in accordance with the manufacturer’s guidelines. In brief, emulsions of cells and beads were created, followed by processes such as reverse transcription, cDNA amplification (with 5 µL of amplified cDNA set aside for targeted scRNA-seq amplification), fragmentation, and ligation with adaptors, along with sample index PCR. Subsequently, barcoding of cells occurred, and a cDNA library was constructed. Sequencing was executed on a NovaSeq 6000 sequencer (Illumina) to obtain paired-end 150 bp reads.

### Quality control and bioinformatics analyses of scRNA-seq data

The Illumina bcl2fastq system (V2.19.1) demultiplexed the sequencing output and converted it into FASTQ format. Cell-distinguishing barcode information and UMI demultiplexing were processed using the parameters recommended by the Cell Ranger pipeline. The *Sus scrofa* genome version Sscrofa11.1 and the PEDV genome (GenBank accession number PV449162.1) were used to align the scRNA-seq data. Filtering and subsequent analyses were performed using the R toolkit Seurat v 4.3.0 in RStudio (v4.4.2). Initial clustering was achieved by accepting cells with less than 30% mitochondrial genes and regressing out the percentage of mitochondrial content and the total number of genes per cell. Cells with fewer than 200 minimal genes and less than 5,800 maximal genes expressed in fewer than three cells were removed to maintain high-quality data sets for downstream analyses.

Following the quality control of the obtained cells, we conducted dimensionality reduction, and the outcomes were visualized using t-distributed stochastic neighbor embedding (tSNE). To achieve this, the logNormalize "normalized" function was employed to calculate the expression values for each gene, and the RunPCA algorithm was applied to the normalized gene expression lists. The t-SNE of the data set was computed using the top 20 ranked principal components (PCs). Cells underwent clustering through a shared nearest-neighbor (SNN) graph-based method. Subsequently, each cluster was scrutinized for differentially expressed genes using the FindAllMarkers function to categorize them based on highly expressed genes. The cell type of the initial cluster was identified by assessing the average expression of marker genes indicative of various intestinal cell types.

### Cell–cell communication analysis

Cell-cell communication analysis was performed using CellChat v1.6.1, which infers intercellular interactions based on ligand-receptor signaling with default parameters. The “netVisual_diffInteraction” function was used to visualize differences in the strength of cell-cell communication.

### Single-cell pseudo-time trajectory analysis

Monocle 2 (version 2.24.1) was used to analyze differential gene expression patterns along the trajectory of cell state transitions. Pseudotime analysis of B cells was performed using the top 100 DEGs in B cell subtypes, identified by Seurat. Dimensionality reduction was conducted with the DDRTree algorithm, and the “orderCells” function was applied to reconstruct the cell state trajectory.

### Isolation of porcine peripheral blood mononuclear cells (PBMCs)

PBMCs were isolated via the precaval vein from the blood of 5- to 8-week-old swine. The blood was diluted with an equal volume of sterile phosphate-buffered saline (PBS) and carefully layered onto Histopaque-1077 (Sigma-Aldrich) before being centrifuged at 800 × *g* for 25 min. Following centrifugation, the middle white mist-like layer containing mononuclear cells was carefully collected and subjected to an additional centrifugation step at 600 × *g* for 15 min. The resulting cell pellet was resuspended in red blood cell lysis buffer (Beyotime) and incubated for 5 min to remove residual erythrocytes. After lysis, the cells were centrifuged at 600 × *g* for 6 min, washed twice with PBS, and centrifuged again under the same conditions. The purified PBMCs were resuspended in pre-warmed Roswell Park Memorial Institute-1640 (RPMI-1640) medium supplemented with 1 U/mL penicillin and streptomycin, then seeded at a density of 10⁶ cells/mL in six-well plates for subsequent experiments.

### Flow cytometry

PBMCs were co-incubated with PEDV in culture medium containing 6 µg/mL trypsin for 24 h. Following incubation, the cells were harvested by centrifugation at 600 × *g* for 10 min and resuspended in 500 µL of PBS. Surface marker-specific antibodies were added to the suspension, and the cells were incubated at 4°C for 30 min. After incubation, the cells were washed three times with PBS. Permeabilization and fixation were performed using the BD Cytofix/Cytoperm Fixation/Permeabilization Kit (BD Biosciences) according to the manufacturer’s instructions, followed by three washes with wash buffer. The cells were then incubated with antibodies targeting dsRNA and the PEDV S protein at 4°C for 30 min. After three additional PBS washes, each for 5 min, the cell pellet was resuspended and analyzed by flow cytometry. The apoptosis of T cells was measured by One-step TUNEL Cy5 Apoptosis Detection Kit (APExBIO) following protocols recommended by the manufacturer. The data from flow cytometry were analyzed using FlowJo vX.

### qRT–PCR

IPI-2I (porcine ileal epithelial cells) cells were infected with PEDV at a multiplicity of infection (MOI) of 0.1. At 24 h post-infection, cell samples were collected for RNA extraction. Total RNA was extracted using a total RNA kit and reverse transcribed into cDNA following the manufacturer’s instructions. Real-time PCR was performed with FastStart Universal SYBR Green Master. The expression levels of target gene mRNAs were quantified using specific primers listed in Table S2. Each mRNA transcript was analyzed in triplicate and normalized to the expression of the housekeeping gene β-actin.

### Statistical analysis

Each experiment was performed at least three times. Statistical significance was assessed using two-way ANOVA. Data analysis was conducted with GraphPad Prism v9, with *P*-values < 0.05 considered statistically significant and *P*-values < 0.01 regarded as highly significant.

## Data Availability

The authors confirm that the data supporting the findings of this study are available within the article and its supplemental material. The single-cell RNA sequencing data set generated in this study has been deposited in the NCBI Gene Expression Omnibus (GEO) under accession number GSE293713.

## SUPPLEMENTAL MATERIAL

Supplemental material jvi.00555-25-s0001.docx Tables S1 and S2; Fig. S1 to S4.

ASM does not own the copyrights to Supplemental Material that may be linked to, or accessed through, an article. The authors have granted ASM a non-exclusive, world-wide license to publish the Supplemental Material files. Please contact the corresponding author directly for reuse.
