# Supplementary material for: Unraveling the cross-talk between a highly virulent PEDV strain and the host via single-cell transcriptomic analysis
Source: J Virol. 2025 May 21;99(6):e00555-25. doi: 10.1128/jvi.00555-25 (PMC12172440; doi:10.1128/jvi.00555-25)
Supplement: Supplemental material — Tables S1 and S2; Fig. S1 to S4. [file jvi.00555-25-s0001.docx]

**Table S1. PDEV reference strains used for phylogenetic analysis in this study.**

| Strain name | Year | Place of isolation | Genbank Accession Number |
| --- | --- | --- | --- |
| AJ1102 | 2011 | China | JX188454.1 |
| DR13 | 1999 | South Korea | JQ023162.1 |
| BJ-2011-1 | 2011 | China | [JN825712.1](https://www.ncbi.nlm.nih.gov/nuccore/JN825712.1) |
| CH-SCAY-1-2015 | 2015 | China | KU975403.1 |
| CH/GX/2015/750A | 2015 | China | [KY793536.1](https://www.ncbi.nlm.nih.gov/nuccore/KY793536.1) |
| CH/GZSB/05/2020 | 2020 | China | MZ161080.1 |
| CH/HNLH/2015 | 2015 | China | [KT199103.1](https://www.ncbi.nlm.nih.gov/nuccore/KT199103.1) |
| CH/JX-1/2013 | 2013 | China | [KF760557.2](https://www.ncbi.nlm.nih.gov/nuccore/KF760557.2) |
| CH/JXJA/2017 | 2017 | China | [MF375374.](https://www.ncbi.nlm.nih.gov/nuccore/MF375374.1)1 |
| CHM2013 | 2013 | China | [KM887144.1](https://www.ncbi.nlm.nih.gov/nuccore/KM887144.1) |
| CH/S | 1986 | China | [JN547228.1](https://www.ncbi.nlm.nih.gov/nuccore/JN547228.1) |
| CH/SCMY/2018 | 2018 | China | [MH061343.1](https://www.ncbi.nlm.nih.gov/nuccore/MH061343.1) |
| CH/SCQX1/2017 | 2017 | China | MH053416.1 |
| CH/SCST/04/2020 | 2020 | China | MZ161081.1 |
| CH/SCZG/2017 | 2017 | China | [MH061337.1](https://www.ncbi.nlm.nih.gov/nuccore/MH061337.1) |
| CH/SCZY103/2017 | 2017 | China | [MH061340.1](https://www.ncbi.nlm.nih.gov/nuccore/MH061340.1) |
| CH/SCZY44/2017 | 2017 | China | [MH061338.1](https://www.ncbi.nlm.nih.gov/nuccore/MH061338.1) |
| CH/YNKM-8/2013 | 2013 | China | AHG97485.1 |
| CV777 | 1977 | Belgium | [AF353511.1](https://www.ncbi.nlm.nih.gov/nuccore/AF353511.1) |
| EAS1 | 2014 | Thailand | [KR610991.1](https://www.ncbi.nlm.nih.gov/nuccore/KR610991.1) |
| GD-1 | 2011 | China | [JX647847.1](https://www.ncbi.nlm.nih.gov/nuccore/JX647847.1) |
| GD-A | 2012 | China | [JX112709.1](https://www.ncbi.nlm.nih.gov/nuccore/JX112709.1) |
| GD-B | 2012 | China | [JX088695.1](https://www.ncbi.nlm.nih.gov/nuccore/JX088695.1) |
| GDS01 | 2012 | China | [KM089829.1](https://www.ncbi.nlm.nih.gov/nuccore/KM089829.1) |
| GDS21 | 2014 | China | [MH726371.1](https://www.ncbi.nlm.nih.gov/nuccore/MH726371.1) |
| H11-SD2017 | 2017 | China | [MH708243.1](https://www.ncbi.nlm.nih.gov/nuccore/MH708243.1) |
| CH/JSHY/09/2020 | 2020 | China | [MZ161082.1](https://www.ncbi.nlm.nih.gov/nuccore/MZ161082.1) |
| IA2 | 2013 | USA | [KF468754.1](https://www.ncbi.nlm.nih.gov/nuccore/KF468754.1) |
| IBT-VN | 2018 | Viet Nam | [MT198679.1](https://www.ncbi.nlm.nih.gov/nuccore/MT198679.1) |
| PEDV JS-A | 2017 | China | [MH748550.1](https://www.ncbi.nlm.nih.gov/nuccore/MH748550.1) |
| JS2008 | 2013 | China | [KC109141.1](https://www.ncbi.nlm.nih.gov/nuccore/KC109141.1) |
| KB2013-4 | 2013 | China | [KX580953.1](https://www.ncbi.nlm.nih.gov/nuccore/KX580953.1) |
| KNU-1904 | 2019 | South Korea | [MN971595.1](https://www.ncbi.nlm.nih.gov/nuccore/MN971595.1) |
| LC | 2011 | China | [JX489155.1](https://www.ncbi.nlm.nih.gov/nuccore/JX489155.1) |
| LW/L | 2010 | China | [MK392335.1](https://www.ncbi.nlm.nih.gov/nuccore/MK392335.1) |
| LZC | 2007 | China | [EF185992.1](https://www.ncbi.nlm.nih.gov/nuccore/EF185992.1) |
| NW8 | 2015 | China | [MF782687.1](https://www.ncbi.nlm.nih.gov/nuccore/MF782687.1) |
| OH851 | 2014 | USA | [KJ399978.1](https://www.ncbi.nlm.nih.gov/nuccore/KJ399978.1) |
| PC21A | 2013 | USA | [KR078299.1](https://www.ncbi.nlm.nih.gov/nuccore/KR078299.1) |
| PEDV-LYG | 2014 | China | [KM609212.1](https://www.ncbi.nlm.nih.gov/nuccore/KM609212.1) |
| PPC 14 | 2014 | South Korea | [MG781192.1](https://www.ncbi.nlm.nih.gov/nuccore/MG781192.1) |
| SC1402 | 2014 | China | [KP162057.1](https://www.ncbi.nlm.nih.gov/nuccore/KP162057.1) |
| SD-M | 2012 | China | [JX560761.1](https://www.ncbi.nlm.nih.gov/nuccore/JX560761.1) |
| SM98 | 2011 | China | [GU937797.1](https://www.ncbi.nlm.nih.gov/nuccore/GU937797.1) |
| SXSL | 2020 | China | [MZ241108.1](https://www.ncbi.nlm.nih.gov/nuccore/MZ241108.1) |
| USA/Colorado/2013 | 2013 | USA | [KF272920.1](https://www.ncbi.nlm.nih.gov/nuccore/KF272920.1) |
| USA/Iowa107/2013 | 2013 | USA | [KJ645696.1](https://www.ncbi.nlm.nih.gov/nuccore/KJ645696.1) |
| YC2014 | 2014 | China | [KU252649.1](https://www.ncbi.nlm.nih.gov/nuccore/KU252649.1) |
| YN144 | 2014 | China | [KT021232.1](https://www.ncbi.nlm.nih.gov/nuccore/KT021232.1) |
| YN15 | 2013 | China | [KT021228.1](https://www.ncbi.nlm.nih.gov/nuccore/KT021228.1) |
| YN30 | 2013 | China | [KT021229.1](https://www.ncbi.nlm.nih.gov/nuccore/KT021229.1) |
| ZJCZ4 | 2011 | China | [JX524137.1](https://www.ncbi.nlm.nih.gov/nuccore/JX524137.1) |
| ZJU/G1/2013 | 2013 | China | [KU664503.1](https://www.ncbi.nlm.nih.gov/nuccore/KU664503.1) |

**Table S2. The primers used in this study.**

| Target | Primer Sequence (5′ - 3′) |
| --- | --- |
| PEDV-365-F | GGGTGCCATTATCCCTCTATGC |
| PEDV-365-R | AATCTCAACTACACTCGGGAGC |
| PoRV-F | CCGTCATCATCATCCTTGAAT |
| PoRV-R | CTAAAAGCGGTAATGTCACTG |
| TGEV-N-F | AACACGTGGTCGCTCCAA |
| TGEV-N-R | AGCACCACGACTACCAAG |
| PDCoV N-F | CCCCAACAATCCTAAACATCA |
| PDCoV N-R | TCATAGTCGGGAGAACCCTC |
| PEDV-758-F | GAGTCGTGGTAATGGCAACA |
| PEDV-758-R | AGATGGCCTCTTCATTCAGC |
| PEDV-sgN-F | ATCGCAAGTGCTGTGCTG |
| PEDV-sgN-R | CGTTTGCGGCCACGATCC |
| β-actin-F | GGTGGGTATGGGTCAGAAAG |
| β-actin-R | TCCATGTCGTCCCAGTTGGT |
| ISG15-F | GAGCTCAAGCAGCAGGTGTG |
| ISG15-R | CCGCAGGCGCAGATTCATAT |
| STAT1-F | AGGTTCATCAGCTCTACGATGAC |
| STAT1-R | AAAATCGACTGTACTGATCATCC |
| STAT2-F | CAGGAACTGACAACGGACACC |
| STAT2-R | GGGAGGAGAACTGCCAACTGA |
| STAT3-F | AGCAGAAAGTGAGCTACAAAGGG |
| STAT3-R | TTGTAAACTGGACACCGGTCTTG |
| IL18-F | AATCGGATTACTTTGGCAAG |
| IL18-R | ACTGCCAGACCTCTAGTGAG |
| JAK1-F | ACAAGACCATTTGTGACAGT |
| JAK1-R | CCGTCATTTGGATGAAACCG |
| IFIT2-F | TGCCAAATAATGCCCACC |
| IFIT2-R | TAGGAGCAGACAAGGAACAAAT |
| DDX60-F | TGCTCGATGGAGACTCCTTG |
| DDX60-R | GGGAGAAGTTCAGGCAAGTT |
| NFKBIA-F | CGAGGACGGAGACTCGTT |
| NFKBIA-R | GCCAGCTTCCAGAAGTGC |
| KLF6-F | GACTGCTGCGCTCCCGAC |
| KLF6-R | ACGCTCCAACTCCAGGCA |
| MAP3K8-F | TGGACCCATGAGAGAATT |
| MAP3K8-R | GCCCCCAGGCTGTAGATG |
| IRF7-F | AAGAGCCTCGTCCTGGTG |
| IRF7-R | TCCAGCTCGTCATAGAGG |


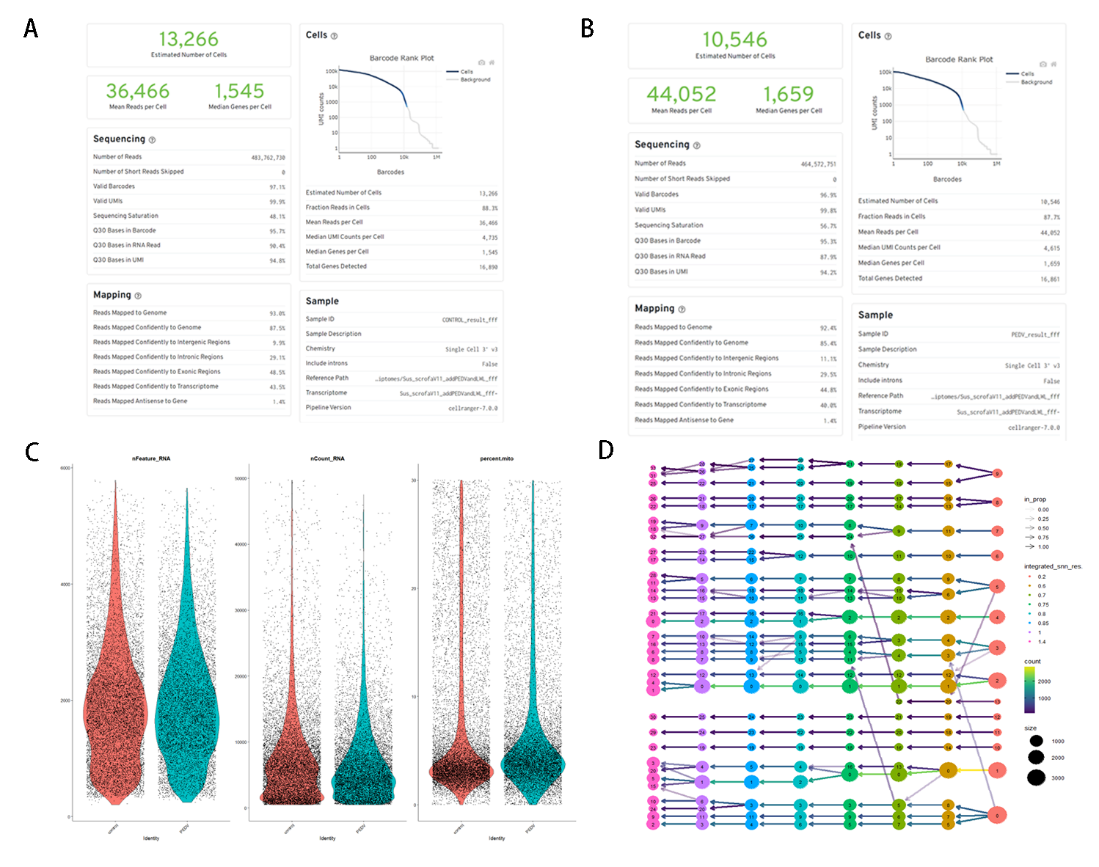


**FIG S1 Quality control overview of sample cells.** (A) Statistical results of PEDV-infected group cell quality. (B) Statistical results of control group sample cell quality. (C) The violin plot shows the number of genes detected for all cells, the number of gene sequences, and the proportion of mitochondria in the cells of the samples respectively. (D) Clustree was used to visualize cell clusters at multiple resolutions.


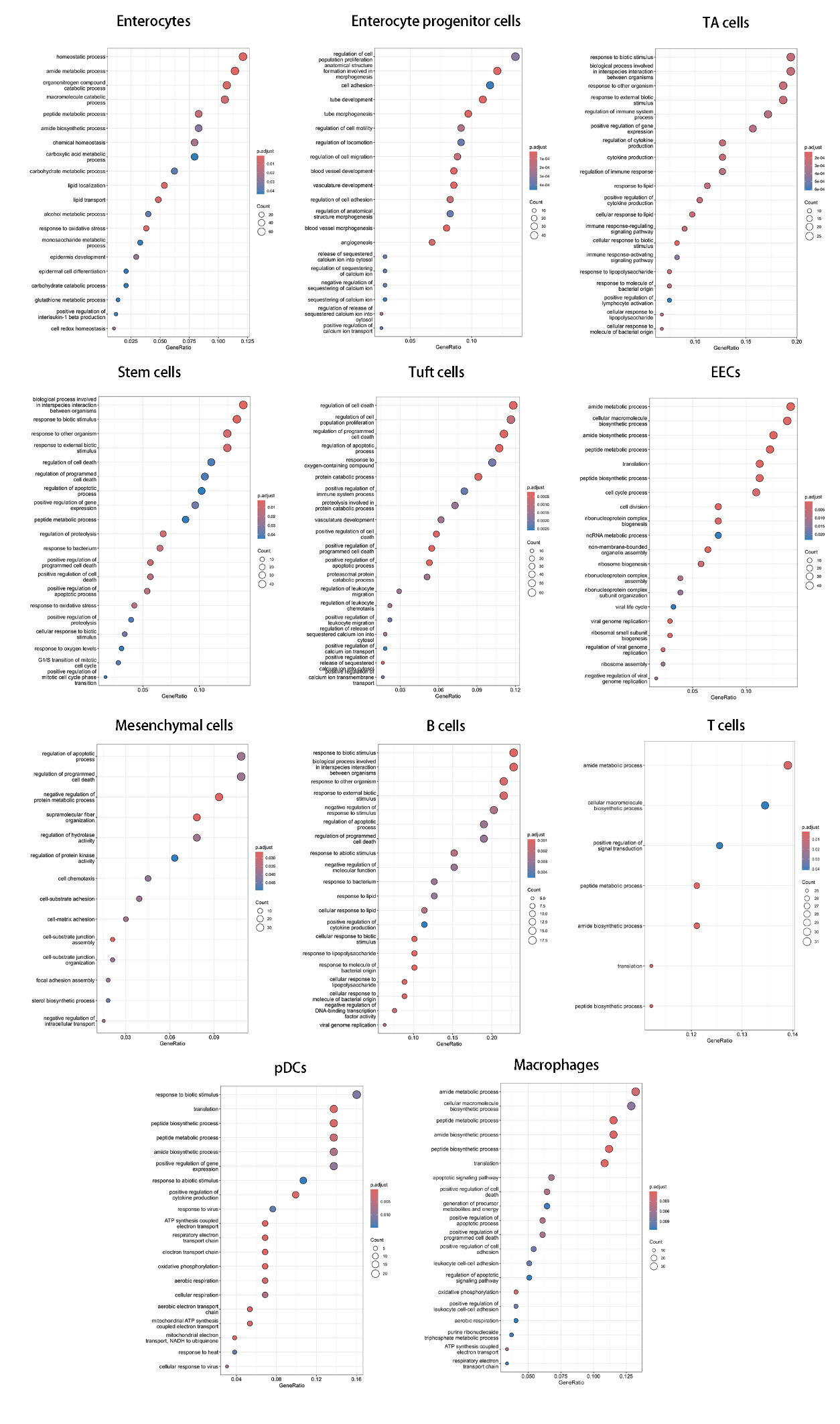


**FIG S2 Transcriptomic characteristics between infected and control intestine.**

Dot plots displaying the top 20 enriched GO terms of differentially expressed genes (DEGs) in the identified cell types (*p*<0.05). Dot color represents the statistical significance of the GO terms, while dot size indicates the number of genes associated with each term.


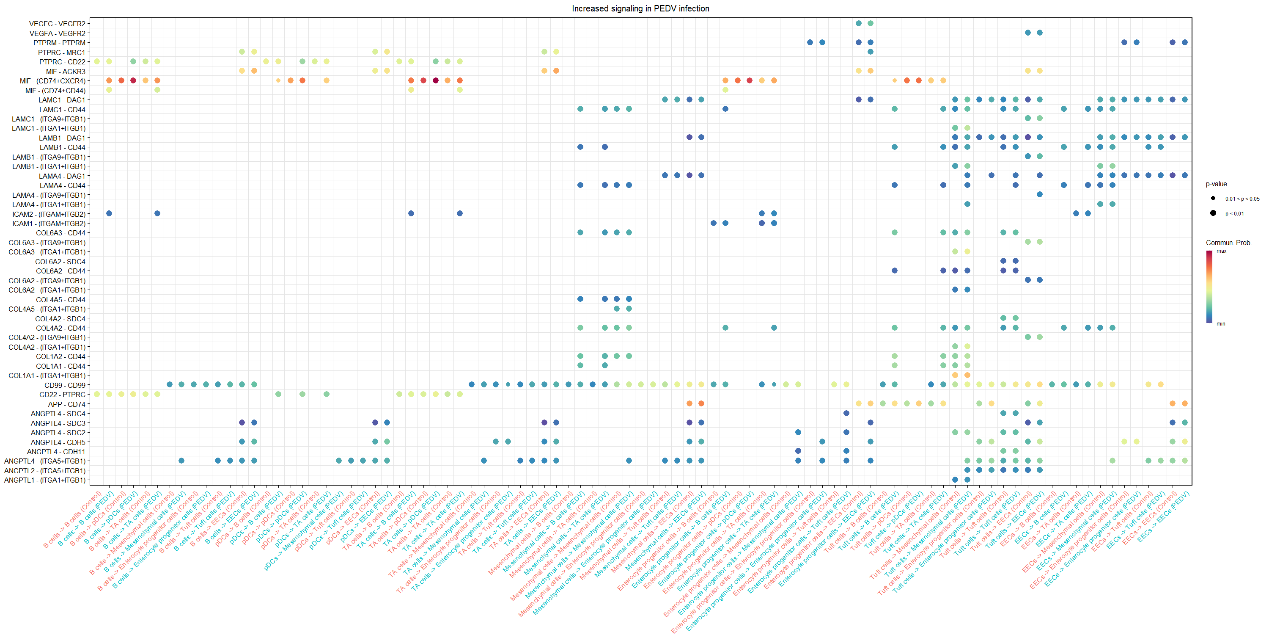


**FIG S3 Comparison of significant ligand-receptor pairs between control and PEDV-infected groups.**

Dot color reflects communication probabilities and dot size represents computed p-values. Empty space means the communication probability is zero. *p*-values are computed from one-sided permutation test.


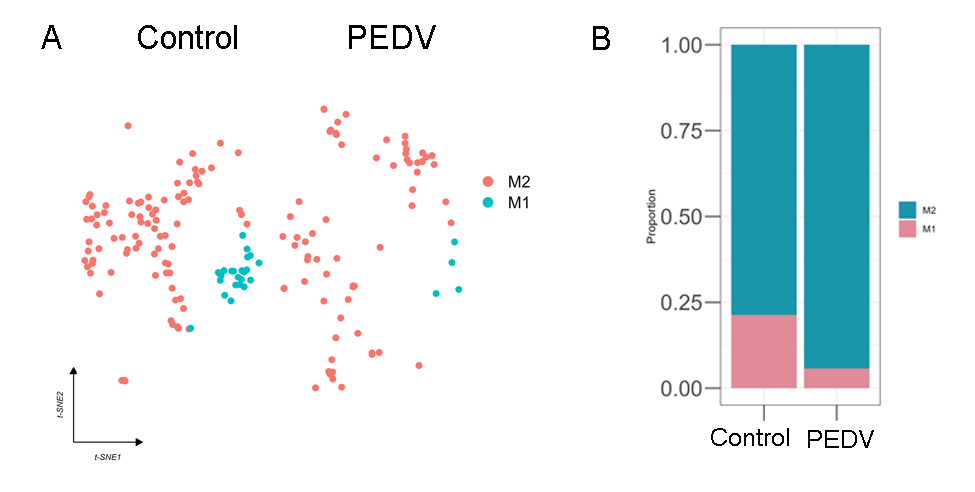


**FIG S4 Subtype analysis of macrophages.**

(A) t-SNE plot showing the classification of macrophages into M1 (blue) and M2 (red) subtypes in control and PEDV-infected samples based on marker gene expression. (B) Bar plot comparing the proportion of M1 and M2 macrophages between control and PEDV-infected groups.
